# A Novel Robot-Aided Upper Limb Rehabilitation Training System Based on Multimodal Feedback

**DOI:** 10.3389/frobt.2019.00102

**Published:** 2019-11-08

**Authors:** Lizheng Pan, Lu Zhao, Aiguo Song, Zeming Yin, Shigang She

**Affiliations:** ^1^School of Mechanical Engineering, Changzhou University, Changzhou, China; ^2^Remote Measurement and Control Key Lab of Jiangsu Province, School of Instrument Science and Engineering, Southeast University, Nanjing, China

**Keywords:** rehabilitation robot, upper limb, motion training, multimodal feedback, stroke

## Abstract

During robot-aided rehabilitation exercises, monotonous, and repetitive actions can, to the subject, feel tedious and tiring, so improving the subject's motivation and active participation in the training is very important. A novel robot-aided upper limb rehabilitation training system, based on multimodal feedback, is proposed in this investigation. To increase the subject's interest and participation, a friendly graphical user interface and diversiform game-based rehabilitation training tasks incorporating multimodal feedback are designed, to provide the subject with colorful and engaging motor training. During this training, appropriate visual, auditory, and tactile feedback is employed to improve the subject's motivation via multi-sensory incentives relevant to the training performance. This approach is similar to methods applied by physiotherapists to keep the subject focused on motor training tasks. The experimental results verify the effectiveness of the designed multimodal feedback strategy in promoting the subject's participation and motivation.

## Introduction

Strokes are caused by acute cerebrovascular disease in one of the cerebral hemispheres, usually associating with impairment of the motor functions and other functional disabilities. Hemiplegic is the most common outcome of a stroke (Guo et al., 2017). According to the statistics of the “high-risk population screening and intervention program for strokes” (Wang et al., 2018), the prevalence rate of this type of brain impairment increased from 1.89% in 2012 to 2.19% in 2016. By this calculation, it can be inferred that the number of stroke patients aged 40 and above in China has now reached 12.42 million. Approximately 80–90% of stroke patients suffer from some form of dysfunction, of which the incidence of upper limb dysfunction is as high as 80% (Fernandes et al., 2018; Sun et al., 2018). This disease has a slow recovery rate and is accompanied by varying degrees of dysfunction that affect patients' lives and imposes a considerable burden on their families (Michael et al., 2005; Edwards et al., 2006). Stroke patients are eager to obtain systematical rehabilitation treatment and return to healthy lives.

Medical theory and clinical practice have proved that the central nervous system of the human brain has a high degree of plasticity (Sano and Ishii, 1978). Neuronal plasticity (Kwakkel et al., 2008) opens up many possibilities for the rehabilitation of hemiplegic patients. According to the theory, the “model integration” of the cerebral cortex functional areas is achieved by inputting regular motions, as the repeated movements can improve motor coordination. The motion of muscles and joints also provides a large number of stimulations to the central nervous system of the brain, which can effectively prevent limb paralysis and muscle atrophy. However, conventional hand-in-hand rehabilitation has many drawbacks, such as a limited number of therapists, high treatment costs, long duration, and tiring training processes. Furthermore, the lack of accurate, objective evaluation mechanisms and real-time feedback of training statuses are urgent problems to be solved, which to some extent stunt the progress of treatment.

To provide immediate and appropriate treatment for stroke patients, many research institutes throughout the world have adopted robot technology (Ploughman and Corbett, 2004) and virtual reality (Saposnik et al., 2016) to help stroke patients perform rehabilitation training tasks. Substantial progress has been made, such as MIT-Manus (Volpe et al., 2000, 2003), GENTLE/S (Schmidt et al., 2004), and RUPERT (Balasubramanian et al., 2008). To make the rehabilitation process more interesting, domestic and international researchers are integrating toys and games into the design of robot-assisted rehabilitation systems. Experiments show that the application of toys and games invokes in patients feelings of pleasure, competition, and other emotions. This can encourage patients to more actively participate in rehabilitation training, resulting in longer training periods and better training results (Fluet et al., 2012; Bank et al., 2018; Avola et al., 2019). Ustinova et al. (2010) and Li et al. (2014) explored the influence of a virtual environment on arm stretching exercises, in which participants were asked to perform simulations of certain daily activities, such as gardening, shopping, and washing clothes. The results showed that patients had a stronger sense of active participation and clearer treatment objectives. Weiss et al. (2004) provided users with a virtual participatory environment, constructing various game scenes and adding corresponding auditory feedback, which enabled patients to maintain long-term interest. In addition, motion estimation (Jurgen et al., 2007), force feedback (Gorsic et al., 2017) and electrical stimulation (Berenpas et al., 2019; Li et al., 2019) are employed in rehabilitation training studies. However, current research usually applies only audio-visual or relatively limited feedback, which struggles to satisfy the requirements of personalized and intelligent rehabilitation training programs (Carignan et al., 2005; Shin et al., 2014).

To provide a more effective robot-aided rehabilitation training, a novel game-based training task with multi-modal feedback strategy is proposed to develop a more humanized training system. The rehabilitation system designed on the basis of the proposed method provides the subject with multi-sensory (visual, auditory, and tactile) feedback. During motor training, a variety of feedback is employed to help patients enjoy training, improving their motivation and active participation in the tasks.

## Materials and Methods

### Background

Clinical studies demonstrate that functional motor training plays a key role in the recovery of the central nervous system after a stroke. In general, rehabilitation training methods are primarily divided into four types: passive, assisted-active, active, and resistance. Patients experiencing paralysis and spasms are unable to make any active movements, thus passive training is suitable. Robot-assisted passive movement can enhance motor proprioception, stimulate flexion and extension reflexes, and gradually increase the range of motions available to joints. In assisted-active training, the rehabilitation robot limits abnormal motions and provides appropriate real-time assistance to the patient as they sense the state of their limbs. In active training processes, patients do not need auxiliary force or external resistance, and the entire training exercise is completed by them actively contracting their upper limb muscles, which can stimulate their active training consciousness and helps them maintain control of their nervous system. To maximize the motor function of the affected limbs, further muscle exercise and resistance training are usually adopted to enhance muscle strength and motor coordination. In our investigation, the designed rehabilitation system is based on the proposed game-based training tasks, and multi-modal feedback provides the subject with active training results.

### Rehabilitation Training System Setup

**Figure 1:**
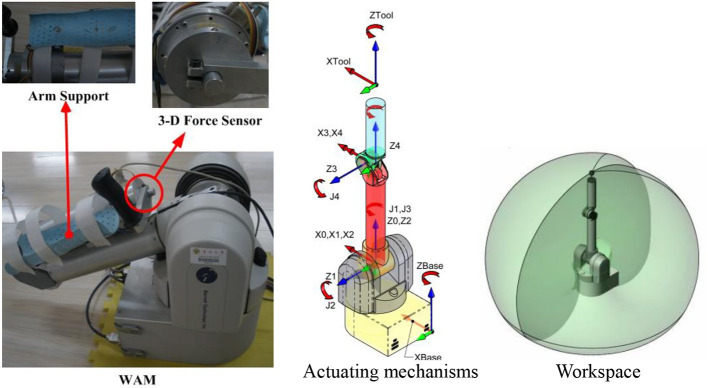
WAM rehabilitation robot.

**Figure 2:**
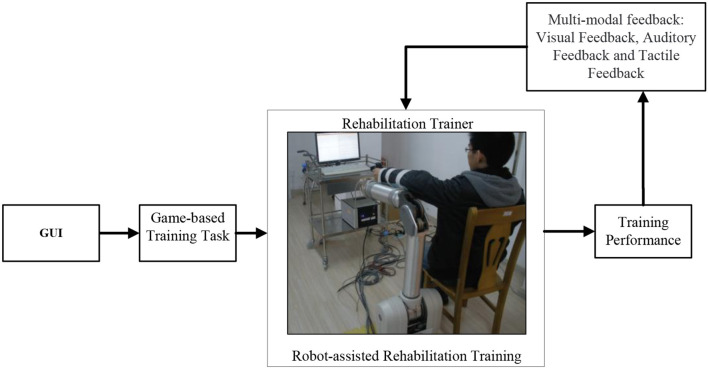
Framework of rehabilitation control system based on multimodal feedback.

In our previous research, a robot-aided upper limb rehabilitation system was constructed, which primarily included the whole arm manipulator (WAM), arm support device, self-developed three-dimensional (3D) force sensor, and controlling personal computer (PC) (Pan et al., 2017, 2019). The WAM works in a large workspace with four rotational degrees of freedom, and the self-developed 3D force sensor is installed at the endpoint of the WAM to measure the interactive force for use in some of the designed control algorithms, as shown in Figure 1. During operation, four driver motor angles can be measured to detect the position of every joint in real time, and the control torque can be set to provide joint control. For detailed information about the hardware and software characteristics of the constructed motion rehabilitation training system, please refer to Pan et al. (2017, 2019). The framework of the rehabilitation control system in this study, which incorporates multimodal feedback, is presented in Figure 2. The subject selects the appropriate game-based training task via a graphical user interface (GUI) to commence the training. The subject controls the end position of the robot arm determining the movement of the mouse, which they operate with their upper limbs, to conduct the game-based motion rehabilitation training. During the training, multimodal feedback is applied to the subject according to the motor performance, to improve the subject's training motivation and participation level.

### GUI and Training Task

#### GUI

**Figure 3:**
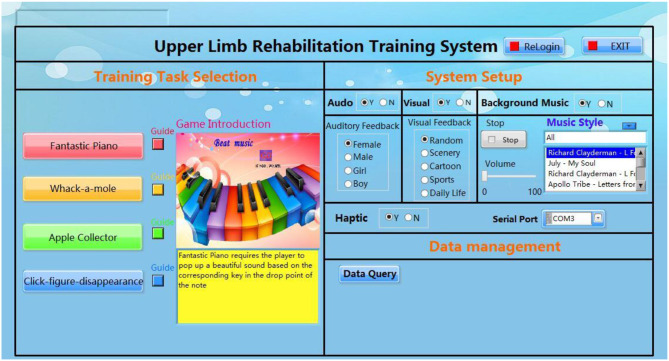
GUI of the upper limb rehabilitation system.

The design of a rehabilitation system should be humanized and user-sensitive. Figure 3 shows the GUI of the upper limb rehabilitation training system. The interface is predominantly blue since blue is typically associated with feelings of comfort and calm. The GUI consists of three main components: training task selection, system setup, and data management. In the “training task” component, users can familiarize themselves with the training tasks using the guidelines and select one of the four tasks provided, according to their preferences. The “system setup” stage allows the user to select their background music and feedback preferences. The background music consists of eight types of music. The feedback options include three types of feedback: visual, auditory, and tactile. During the training process, the relevant experimental data are recorded in real time.

#### Training Task

Currently, the system provides four different types of training task: Fantastic Piano, Apple Collector, Whack-a-mole, and Click-figure-disappearance.

**Figure 4:**
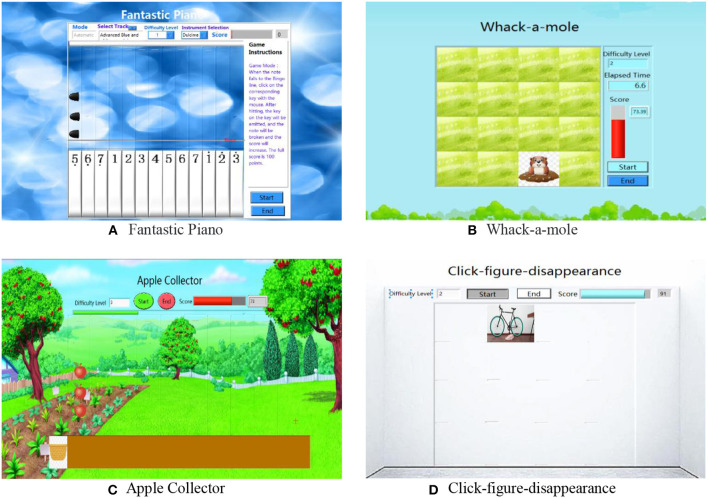
Game-based training tasks. **(A)** Fantastic piano, **(B)** Whack-a-mole, **(C)** Apple Collector, and **(D)** Click-figure-disappearance.

Fantastic Piano (Figure 4A) requires users to move the robot arm and quickly click on the corresponding button according to the drop of a note. When the note falls to the bingo line and the user successfully clicks on the corresponding key, the note is broken, and the score increases. During the rehabilitation training, the subject can view their current score and progress on the task interface. To satisfy the requirements of users of different ages, various tracks are adopted in the system, including “Small Stars,” “Two Tigers,” “Farewell,” “The Marriage of a Fairy Princess,” and “Blue and White Porcelain.”

Whack-a-mole (Figure 4B) requires users to quickly click on a gopher as it randomly appears in the middle lawn, before it disappears again. The performance score depends on the response time and the number of successful hits. The task is divided into three levels; the higher the level, the more challenging it is. With an increase in the level, the specified time that the gopher appears for will decrease.

In Apple Collector (Figure 4C) the user is required to pick up apples, which fall randomly from the sky. Users can set the difficulty of the task prior to starting it. When the number and speed of the falling apples are increased, baskets need to be more quickly and sensitively moved.

The Click-figure-disappearance (Figure 4D) task is designed to train the speed and accuracy of the patient's upper limb movement. When the game starts, various pictures randomly appear on the game interface. If the user clicks on the picture, the picture will disappear; otherwise, the picture remains. When the game interface area is full of pictures, the game ends. The game is also designed with three levels: easy, middle, and high.

#### Data Management

Experimental data are recorded in real time for all training tasks, for future analysis or use in a subsequent session. A temporary file is automatically created before each training session and saved in a specific database. The saved data include the performed rehabilitation motion, task type, level of task, feedback, and score. When users need to query data via the data management module, they are required to enter patient number and training date to obtain the corresponding data.

### Multimodal Feedback

The purpose of applying different feedback modes is to make the training process more interesting, and to motivate patients to give more time and attention to the tasks. During the training process, the system provides the patient with feedback appropriate to their performance. Stimulation includes pictures, complimentary utterances, and touch applied to the arm.

Multimodal feedback comprises three parts: visual, auditory, and tactile feedback. The three types of feedback can be separately employed or applied in combinations, to provide trainees with a variety of options, this is conducive to improving the patient's motivation levels and increasing training efficacy.

#### Visual Feedback

Vision is an important way in which humans experience the world. In the process of rehabilitation training, rich visual stimulation can reduce boredom effectively. We use a variety of images to give more expressive visual feedback, these fall into five categories: “Freedom,” “Landscape,” “Cartoon,” “Sports,” and “Life,” and each category comprises 20 pictures. When the patient's score falls within the range set for the system, pictures will randomly appear, to encourage or praise the user. Thus, the trainee has a more immersive experience and can engage with the game over longer periods.

#### Auditory Feedback

Auditory feedback is an effective means of supplementing visual feedback. In the game, rich and varied sound feedback can provide players with a sense of enjoyment, which renders the game more attractive, and, in terms of rehabilitation, more effective. Users can select their preferences in the GUI and choose the feedback voice characteristics from options such as female voice, male voice, little boy voice, and little girl voice. The auditory feedback is also rich and mainly includes encouraging and suggestive words such as “Come on,” “Work hard,” “Great,” and so on.

#### Haptic Feedback

Haptic sensation is a tactile experience that cannot be obtained through visual or auditory methods. During a long and tedious rehabilitation training session, patients may become bored and lose interest in the training task. At this point, proper tactile stimulation helps the subject to regain their focus on the task. A humanized design principle is employed, prioritizing the comfort needs of patients. In this study, a small, commonly utilized massager, which has a small size, low weight, low power consumption, and adjustable current, is adopted. The massager can be easily adapted to the patients' specific situation.

During the game, if the three kinds of feedback are always active simultaneously, the game will become monotonous, which may leave the patient prone to disengagement. In this research, the multimodal feedback strategy is proposed to improve the subject's motivation levels and is designed to provide a humanized supply of different feedback types according to the training performance of the subject. According to the subject's sensitivity to pictures, auditory, and tactile stimuli, and by combining the experimental analysis results, adjustment rules for the appearance of visual, auditory, and tactile feedback can be found, as shown in Table 1. For example, auditory, visual, and tactile feedback are all active when the subject performs the game-based task with a high score over HS1 for a period of time T1, HS1, and T1 represent the designated score and time, respectively, and vary with the type of task and level of difficulty.

**Table 1:** Distribution of auditory, visual, and tactile feedback.

	**Low score for a certain time**	**High score for a certain time**	**Low score temporarily**	**High score temporarily**	**Generally**
Auditory feedback	✓	✓		✓	✓
Visual feedback	✓	✓	✓		✓
Tactile feedback	✓	✓	✓	✓	

## Experiments

### Experiment Planning

**Figure 5:**
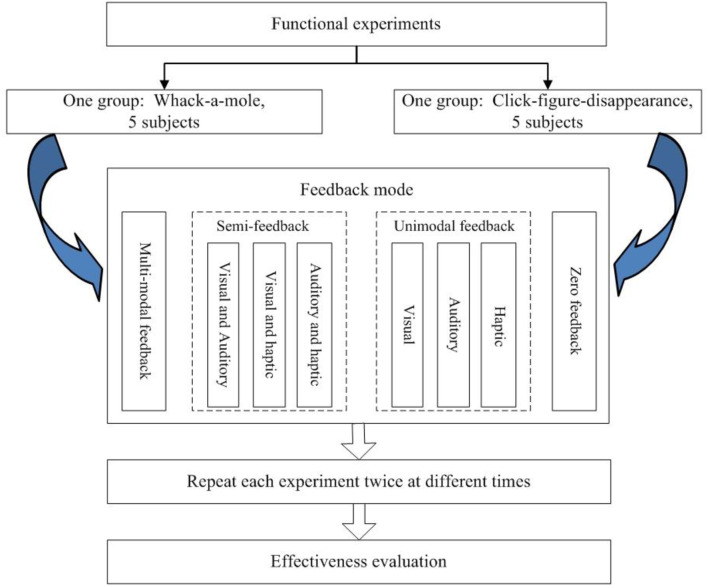
Experiment planning.

To verify the effectiveness of our system in improving the subject's training motivation and active participation, experiments on the functionality of the multimodal feedback strategy were planned and carried out for healthy adults. In order to comprehensively evaluate the performance of the proposed feedback strategy, two different training tasks, Whack-a-mole and Click-figure-disappearance, were selected for verification of the universality of the proposed method. Ten healthy adults were recruited, aged between 23 and 27 years old. Information about the subjects is provided in Table 2. The ten subjects were randomly divided into two groups, one group carrying out experiments with the Whack-a-mole game-based task, the other carrying out experiments with the Click-figure-disappearance game-based task. In order to broadly analyze the effectiveness of different feedback methods on the training performance of subjects, four types of feedback, namely, multimodal feedback (combination of visual, auditory, and tactile feedback), semi-feedback (visual, auditory, and tactile feedback in pair-combinations), unimodal feedback (exclusively visual, auditory or tactile feedback), and zero feedback (without any feedback), were employed. Every subject carried out experiments with each of the eight different feedback types. To ensure the effectiveness of the experiment, the order in which they tested under these different feedback conditions was randomized. Furthermore, each feedback setting test was repeated at other times, to prevent the influence of external, accidental factors. The experiment plan is shown in Figure 5.

**Table 2:** Volunteer information.

**Volunteer**	**Age (years)**	**Gender**
P1	24	Male
P2	25	Female
P3	25	Male
P4	27	Male
P5	24	Female
P6	24	Male
P7	23	Female
P8	24	Male
P9	26	Male
P10	25	Female

### Results and Discussion

In this investigation, the game-based training task and multimodal feedback mechanism are developed to improve the subject's training motivation and active participation level. Appropriate sensory feedback can increase the subject's sense of interaction and thus motivate them. In this research, we investigate the effectiveness of the designed multimodal feedback system in increasing motivation in the subject; all experimental results are analyzed from this perspective.

The average scores of five healthy subjects for the Whack-a-mole game, under different feedback modes, are shown in Table 3. The average scores for the various semi-feedback (pair-combinations of vision, hearing, and touch) options show no significant difference between them, which indicates that when two kinds of feedback are combined, regardless of which pair-combination is used, minimal differences are observed in the improvement in training motivation. The average scores with unimodal feedback (only one feedback occurring) are also not significantly different, that is, there is no evidence suggesting which out of visual, auditory or tactile feedback is more advantageous to the improvement of the subject's training motivation and active participation. Comparing with multimodal feedback, we see that in the first run of experiments the average score of the five subjects under the semi-feedback mode was 85.54, 4.26 lower than that achieved under the multimodal feedback mode, 2.17 higher than that seen under the unimodal feedback mode, and 5.19 higher than under the zero feedback mode. In the second run of experiments, the average score of five subjects under the unimodal feedback mode was 82.91, which is 1.96 lower than that under the semi-feedback mode, 6.19 lower than that under multimodal feedback and higher than seen under the zero feedback mode by 2.1. Overall, the proposed multimodal feedback strategy can be seen to improve the subject's motivation and participation levels effectively.

**Table 3:** Average scores of five subjects with Whack-a-mole.

**Time**	**Multimodal feedback**	**Semi-feedback**			**Unimodal feedback**			**Zero feedback**
		**Visual-auditory**	**Visual-tactile**	**Auditory-tactile**	**Visual**	**Auditory**	**Tactile**	
1	89.8	85.62	85.07	85.92	82.75	83.87	83.49	80.35
2	89.1	85.24	84.18	85.2	82.85	82.74	83.13	80.81

Experiments were performed with the other group on the Click-figure-disappearance game, to observe the feedback effectiveness across different tasks. The experimental results are summarized in Table 4. The average scores under multimodal feedback in the first experimental run are the highest, 3.17 higher than the average score of 85.62 achieved under the semi-feedback mode, 5.36 higher than the average score of 83.43 found under the unimodal feedback mode, and 5.86 higher than that seen under the zero feedback mode. In general, the greater the amount of sensory feedback, the higher the training performance. Both groups shown the best performance under multimodal feedback.

**Table 4:** Average scores of five subjects with Click-figure-disappearance.

**Time**	**Multimodal feedback**	**Semi-feedback**			**Unimodal feedback**			**Zero feedback**
		**Visual-auditory**	**Visual-tactile**	**Auditory-tactile**	**Visual**	**Auditory**	**Tactile**	
1	88.79	86.28	85.29	85.29	83.45	83.14	83.7	82.64
2	88.35	85.49	85.18	85.12	83.74	83.96	83.40	82.49

Additionally, to analyze and compare from multiple perspectives, Tables 5, 6 show the average scores of the twice-repeated experiments for each subject under various feedback conditions with Whack-a-mole or Click-figure-disappearance. It can be seen by analyzing Tables 5, 6 that the more feedback varies, the better the subject's performance. The experimental results indicate that training with multimodal feedback can enable the subject to maintain a high level of training motivation and improves performance. When employing multimodal feedback during training, the subject is provided with multiple types of multi-sensory feedback, which encourages or praises them, and keeps them in an active, engaged state.

**Table 5:** Average scores of each subject with Whack-a-mole.

**Subject**	**Multimodal feedback**	**Semi-feedback**			**Unimodal feedback**			**Zero feedback**
		**Visual-auditory**	**Visual-tactile**	**Auditory-tactile**	**Visual**	**Auditory**	**Tactile**	
P1	91.01	86.02	85.47	86.58	84.03	83.22	84.12	81.32
P2	89.61	85.69	85.25	86.75	82.42	84.1	83.68	81.25
P3	87.81	85.17	84.42	83.58	82.69	82.18	82.59	80.31
P4	89.5	84.22	83.09	84.61	81.08	81.85	82.89	79.5
P5	89.32	86.08	84.92	86.26	83.77	85.15	83.27	80.54

**Table 6:** Average scores of each subject with Click-figure-disappearance.

**Subject**	**Multimodal feedback**	**Semi-feedback**			**Unimodal feedback**			**Zero feedback**
		**Visual-auditory**	**Visual-tactile**	**Auditory-tactile**	**Visual**	**Auditory**	**Tactile**	
P6	87.54	84.48	83.67	84.78	83.44	83.16	82.28	81.56
P7	90.75	87.05	85.30	87.21	84.29	84.29	83.85	82.92
P8	89.34	87.37	86.01	83.38	83.12	83.35	84.07	82.6
P9	86.31	84.61	84.92	84.92	82.96	83.31	83.34	82.76
P10	88.83	85.945	86.28	85.73	84.18	83.65	84.21	82.99

For further analysis of the performance of the proposed multimodal feedback strategy, depth analysis was conducted by randomly selecting the experimental data of two subjects from each group. The experimental time label (1st or 2nd repetition of the task under a specific feedback method) was also randomly selected, as shown in Table 7, and the experimental results were analyzed in detail.

**Table 7:** The information of subjects selected.

**Subject**	**Task types**	**Experimental time label**
P1	Whack-a-mole	1
P3	Whack-a-mole	1
P7	Click-figure-disappearance	1
P10	Click-figure-disappearance	2

**Figure 6:**
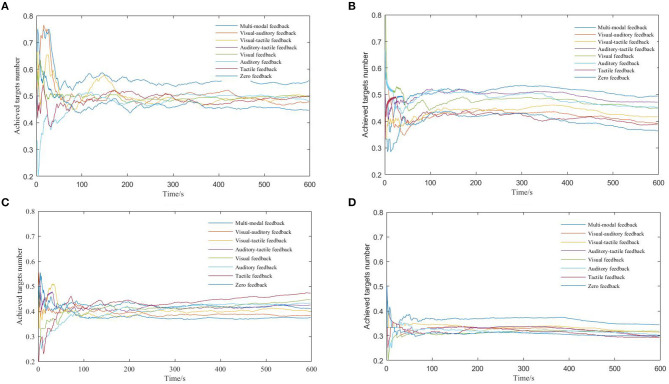
Number of obtained targets per unit time. **(A)** P1, Whack-a-mole, **(B)** P3, Whack-a-mole, **(c)** P7, Click-figure-disappearance, and **(D)** P10, Click-figure-disappearance.

During the game-based training task, the number of goals achieved per unit time is an important indicator reflecting the trainee's performance. In this investigation, 3 s was selected as the unit time; a smooth curve of the number of targets achieved per unit time is presented in Figure 6. Due to the adaptability of the trainee, the operation shows some volatility during the first minute of training. Considering P1 with the Whack-a-mole training task as an example (Figure 6A); the achieved targets per unit time is greater with multimodal feedback than with semi-feedback, unimodal feedback, and zero feedback. There is no distinct difference observed between the three pair-combinations of the semi-feedback mode. The average number of targets achieved per unit time for different feedback modes is presented in Table 8. Considering P1 as an example in Table 8, the average number of obtained targets per unit time for Whack-a-mole is 0.561 when visual, auditory, and tactile feedback are combined, its highest value. For zero feedback, the average number of obtained targets per unit time is lowest, at 0.468. For P1 and P3 with Whack-a-mole, under multimodal feedback, the average number of targets achieved per unit time was 16.6 and 21.2% higher, respectively, than that gained with zero feedback. For P7 and P10 with Click-figure-disappearance, the average number of obtained targets per unit time with multimodal feedback was 9.4 and 14.9% higher, respectively, than that gained with zero feedback. The findings indicate that multimodal feedback can effectively improve the subject training results.

**Table 8:** Average number of obtained targets per unit time.

	**Multimodal feedback**	**Semi-feedback**			**Unimodal feedback**			**Zero feedback**
		**Visual and auditory**	**Visual and tactile**	**Auditory and tactile**	**Visual**	**Auditory**	**Tactile**	
P1, task-1	0.561	0.50	0.499	0.485	0.496	0.480	0.485	0.468
P3, task-1	0.509	0.422	0.435	0.4948	0.476	0.485	0.41	0.401
P7, task-2	0.423	0.393	0.402	0.417	0.413	0.404	0.434	0.383
P10, task-2	0.363	0.327	0.333	0.3288	0.314	0.317	0.313	0.309

*Task-1 represents Whack-a-mole, task-2 represents Click-figure-disappearance*.

**Figure 7:**
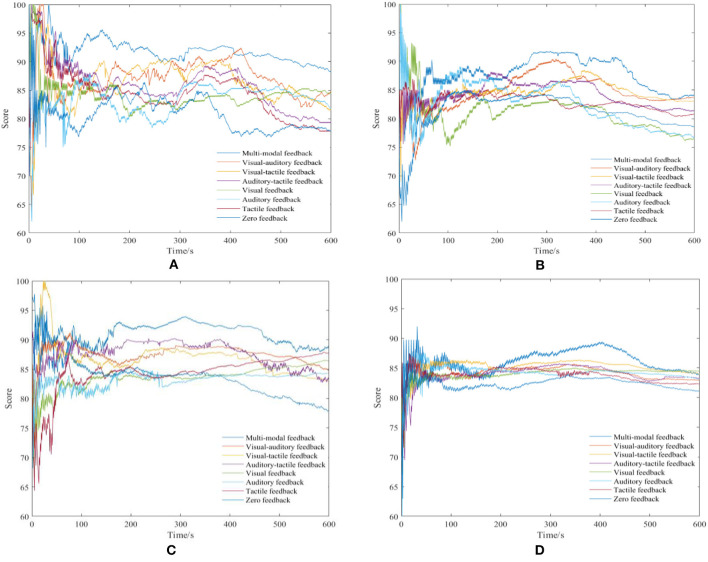
Recorded scores. **(A)** P1, Whack-a-mole, **(B)** P3, Whack-a-mole, **(C)** P7, Click-figure-disappearance, and **(D)** P10, Click-figure-disappearance.

The recorded scores of the four randomly selected subjects are also presented in Figure 7. It can be seen that multimodal feedback best enhances the subject's performance, which is consistent with the conclusions from Tables 3 to 6. As shown in Figure 7, regardless of the feedback form, the results begin to decline after 400 s. Employing sensory feedback can improve participants' motivation and cause subjects to become more actively involved in the training, which is beneficial to the total training performance. However, this result does not change the body itself physiologically, that is, if the training time is sufficiently long, the training motivation and performance of the subject will start gradually to decline. Thus, for motion rehabilitation training, the training time should be set reasonably.

In order to broadly compare the performances of different feedback strategies, all the experimental scores were subjected to further statistical analysis. The independent sample *t*-test was employed to present the statistical analysis within different feedback strategies, and MANOVA was employed to reveal the statistical analysis between feedback type (within subject) and training task (between subject), and *P* < 0. 05 meant that the difference was statistically significant.

In this investigation, four types of feedback, namely, multimodal feedback (combination of visual, auditory, and tactile feedback), semi-feedback (pair-combinations of visual, auditory, and tactile feedback), unimodal feedback (single visual, auditory or tactile feedback), and zero feedback (without any feedback), were employed. Semi-feedback and unimodal feedback both include three feedback patterns. The internal sample *t*-test results for semi- and unimodal feedback with Whack-a-mole are presented in Table 9. They show that there was no statistically significant difference (*P* > 0.05) between the semi-feedback patterns (visual & auditory, visual & tactile, auditory & tactile) or between the unimodal feedback patterns (visual, auditory, tactile), except the comparison of visual-auditory and visual-tactile where the result is *P* < 0.05. It is to say that usually there was no obvious difference in feedback patterns in terms of these types of feedback. Analysis of the four feedback types (multimodal feedback, semi-feedback, unimodal feedback, and zero feedback) was conducted. Single factor analyses of the experimental scores with Whack-a-mole are presented in Table 10. All the analyzed results were *P* < 0.01, presenting extremely statistical significance. It means that the addition of any sensory feedback was effective in improving the training performances, and multimodal feedback worked best.

**Table 9:** Semi-feedback and Unimodal feedback internal *t*-test results for Whack-a-mole.

	**Semi-feedback**			**Unimodal feedback**		
	**VA&VT**	**VA&AT**	**VT&AT**	**V&A**	**V&T**	**A&T**
*t*	5.478	−0.265	−2.068	−1.006	−1.171	−0.018
*P*	0.005	0.803	0.107	0.371	0.306	0.986

*VA, visual and auditory; VT, visual and tactile; AT, auditory and tactile; V, visual feedback; A, auditory feedback; T, tactile feedback*.

**Table 10:** Paired quantitative *t*-test analysis results of four groups of feedback under Whack-a-mole.

	**M&VA**	**M&VT**	**M&AT**	**VA&V**	**VA&A**	**VT&V**	**VT&T**	**AT&A**	**AT&T**	**V&Z**	**A&Z**	**T&Z**
*t*	7.987	9.208	9.801	10.752	5.535	6.364	4.545	5.263	5.675	5.919	5.367	14.24
*P*	0.001	<0.001	<0.001	<0.001	0.005	0.003	0.010	0.006	0.004	0.004	0.005	<0.001

*M, Multimodal feedback; VA, visual and auditory; VT, visual and tactile; AT, auditory and tactile; V, visual feedback; A, auditory feedback; T, tactile feedback; Z, zero feedback*.

The corresponding analysis of the internal patterns of the four feedback types for Click-figure-disappearance is presented in Tables 11, 12. As shown in Table 11, there is no statistically significant difference (*P* > 0.05) between the internal feedback patterns in quantitative *t*-test analysis in terms of unimodal feedback or semi-feedback methods. From Table 12, the analysis results between multimodal feedback, semi-feedback, unimodal feedback, and zero feedback show that the difference in average score was statistically significant (*P* < 0.05) except the comparison of auditory-tactile and tactile. In general, from a statistical point of view, the more sensory feedback modes, the better the training performances. This means that the multimodal feedback can effectively improve the motivation and participation of the trainee.

**Table 11:** Semi-feedback and Unimodal feedback internal *t*-test results for Click-figure-disappearance.

	**Semi-feedback**			**Unimodal feedback**		
	**VA&VT**	**VA&AT**	**VT&AT**	**V&A**	**V&T**	**A&T**
*t*	1.537	0.826	0.041	0.284	0.133	0.006
*P*	0.199	0.454	0.969	0.79	0.9	0.995

*VA, visual and auditory; VT, visual and tactile; AT, auditory and tactile; V, visual feedback; A, auditory feedback; T, tactile feedback*.

**Table 12:** Paired quantitative *t*-test analysis results of four groups of feedback under Click-figure-disappearance.

	**M&VA**	**M&VT**	**M&AT**	**VA&V**	**VA&A**	**VT&V**	**VT&T**	**AT&A**	**AT&T**	**V&Z**	**A&Z**	**T&Z**
*t*	7.261	4.903	4.496	4.082	4.621	3.548	12.454	3.512	2.446	3.427	4.709	6.057
*P*	0.001	0.008	0.010	0.015	0.009	0.023	<0.001	0.024	0.07	0.026	0.009	0.003

*M, Multimodal feedback; VA, visual and auditory; VT, visual and tactile; AT, auditory and tactile; V, visual feedback; A, auditory feedback; T, tactile feedback; Z, zero feedback*.

To analyze the influence of different feedback types and training tasks on trainers' performance, the MANOVA was employed to reveal the statistical analysis with feedback type (within subject) and training task (between subject). The results of statistical analysis were shown in Table 13. It can be seen that there was significant difference in different types of feedback where the *F* = 41.128, *P* = 2.52E-21 < 0.05 was presented. However, there was no statistical difference between the different training tasks when certain feedback methods were used, where *F* = 2.304, *P* = 0.134 > 0.05. It means that the feedback strategy presents good universal applicability. Furthermore, the interaction influence between training tasks and feedback types had no significant difference for training performance as well, where *F* = 1.453, *P* = 0.200 > 0.05. The two-way ANOVA statistical analysis also verified that the more sensory feedback the trainee received, the more motivational the trainee was.

**Table 13:** MANOVA with feedback type and training task.

	**Sum of Squares**	**df**	**Mean square**	***F*-value**	***P*-value**	**F crit**
Tasks	2.758	1	2.759	2.304	0.134	3.990
Feedbacks	344.690	7	49.241	41.128	2.52E-21	2.156
Tasks & feedbacks	12.173	7	1.739	1.453	0.200	2.156
Internal	76.626	64	1.197			
Total	436.248	79				

## Conclusions

Currently, upper-limb rehabilitation training systems are usually designed to serve the subject through specific training exercises with single-sense feedback or without any feedback, which struggles to improve the training motivation of subjects. In this research, a robot-assisted upper limb rehabilitation training system that is based on multimodal feedback is presented. The proposed visual, auditory, and tactile feedback, combined with a game-based training task, is employed to improve the subject's training motivation and active participation. During motion training, the designed rehabilitation system provides subjects with multi-sensory feedback according to their performance and achieves a humanized dynamic feedback interaction. Comparative analysis of different types of functional experiment results show that multimodal feedback can effectively improve the subject's training motivation. In this investigation, we verify the effectiveness of the proposed multimodal feedback strategy in improving motivation. In future research, we will conduct clinical trials with a multimodal feedback strategy for stroke patients, to verify the efficacy.

## Data Availability Statement

The raw data supporting the conclusions of this manuscript will be made available by the authors, without undue reservation, to any qualified researcher.

## Ethics Statement

In this investigation, a novel multimodal feedback strategy is proposed to increase the subject's interest and participation, and the functional experiments were conducted to verify the effectiveness of the designed multimodal feedback strategy. At present, the main purpose is to study the influence of multi-sensory incentives on the participation and training enthusiasm of subjects, which is exempt from ethics approval in accordance with national/institutional guidelines. Meanwhile, before the experiment, the experimental volunteers were informed of the experimental requirements, the experimental form, the data recorded in the experiment, the purpose of the experiment, etc., and agreed to participate in the experiments.

## Author Contributions

LP was responsible for the design of the overall experimental program and paper writing. LZ carried out the experiment and analyzed the collected data. ZY assisted LZ in the experiment and later data analysis. AS gave guidance and revised the paper. SS put forward valuable suggestions on the revision of the paper.

### Conflict of Interest

The authors declare that the research was conducted in the absence of any commercial or financial relationships that could be construed as a potential conflict of interest.
